# Data on the association between a simplified Mediterranean diet score and the incidence of combined, cardio and cerebro vascular events

**DOI:** 10.1016/j.dib.2019.103789

**Published:** 2019-02-28

**Authors:** Fabrizio Veglia, Damiano Baldassarre, Ulf de Faire, Sudhir Kurl, Andries J. Smit, Rainer Rauramaa, Philippe Giral, Mauro Amato, Alessandro Di Minno, Alessio Ravani, Beatrice Frigerio, Samuela Castelnuovo, Daniela Sansaro, Daniela Coggi, Alice Bonomi, Calogero C. Tedesco, Elmo Mannarino, Steve E. Humphries, Anders Hamsten, Elena Tremoli, the IMPROVE study group

**Affiliations:** a Centro Cardiologico Monzino, IRCCS, Milan, Italy; b Department of Medical Biotechnology and Translational Medicine, Università di Milano, Milan, Italy; c Division of Cardiovascular Epidemiology, Institute of Environmental Medicine, Karolinska Institutet, Sweden; d Department of Cardiology, Karolinska University Hospital, Solna, Karolinska Institutet, Stockholm, Sweden; e Institute of Public Health and Clinical Nutrition, University of Eastern Finland, Kuopio Campus, Finland; f Department of Medicine, University Medical Center Groningen, Groningen, the Netherlands; g Foundation for Research in Health Exercise and Nutrition, Kuopio Research Institute of Exercise Medicine, Kuopio, Finland; h Assistance Publique - Hopitaux de Paris, Service Endocrinologie-Metabolisme, Groupe Hôpitalier Pitie-Salpetriere, Unités de Prévention Cardiovasculaire, Paris, France; i Centro Dislipidemie E. Grossi Paoletti, Ospedale Ca' Granda di Niguarda, Milan, Italy; j Dipartimento di Scienze Farmacologiche e Biomolecolari, Università di Milano, Milan, Italy; k Internal Medicine, Angiology and Arteriosclerosis Diseases, Department of Clinical and Experimental Medicine, University of Perugia, Perugia, Italy; l Cardiovascular Genetics, British Heart Foundation Laboratories, Institute Cardiovascular Science, University College of London, Rayne Building, London, United Kingdom; m Atherosclerosis Research Unit, Department of Medicine Solna, Karolinska Institutet, Stockholm, Sweden

## Abstract

Data presented in this article are related to the research article entitled “*A priori-defined Mediterranean-like dietary pattern predicts cardiovascular events better in north Europe than in Mediterranean countries*” [Veglia et al., 2018]. Data contain information about the incidence of cardiovascular events in a high-risk European population (IMPROVE study) [Baldassarre et al., 2010, 2012, 2013]. Combined vascular events, as well as cardio- and cerebro-vascular events were stratified according to a priori-defined simple Mediterranean Diet (MD) score, based on just seven nutritional items (minimal adherence was 0 and maximal adherence was 7).

**Table:** Specifications table

Subject area	Epidemiology
More specific subject area	Cardiovascular prevention; Mediterranean Diet
Type of data	Table and figure
How data was acquired	A dietary questionnaire was administered at baseline by trained personnel. Events were assessed and validated over a 36 month follow-up
Data format	Analyzed
Experimental factors	Data are analyzed to establish the relationship between a simplified MD score and combined, cardio- or cerebrovascular events
Experimental features	Vascular events according to Mediterranean diet Score
Data source location	Kuopio (Finland), Stockholm (Sweden), Groningen (the Netherlands), Paris (France), Milan and Perugia (Italy)
Data accessibility	Data are in this article
Related research article	F. Veglia, D. Baldassarre, U. de Faire, S. Kurl, AJ. Smit, R. Raurama, et al. A priori-defined Mediterranean-like dietary pattern predicts cardiovascular events better in north Europe than in Mediterranean countries. Int J Cardiol. 2018 Nov 29. pii: S0167-5273(18)35681-X. https://doi.org/10.1016/j.ijcard.2018.11.124. [Epub ahead of print] PMID: 30545617

**Table:** 

**Value of the data** • The data on the adherence to the Mediterranean diet using a simple dietary questionnaire, based on a limited number of food items provides a simplified approach that can be used for further investigation on the role of nutritional aspects in the development of cardiovascular pathology. • These data on the association of a priori-defined Mediterranean-like dietary pattern (measured at baseline) with the incidence of combined, cardio- and cerebro-vascular events (VEs) can be used in further studies to compare the adherence to the Mediterranean diet and its effect on cardiovascular diseases in different cohorts.

## 1 Data

Among the 3,703 subjects enrolled in the IMPROVE study [2], [3], [4], 215 (7.96%) developed a first VE: 3 sudden cardiac death, 34 myocardial infarction (7 fatal), 26 hospitalization for angioplasty, 13 coronary bypass grafting, 49 diagnoses of angina pectoris, 32 ischemic stroke (0 fatal), 41 transient ischemic attack, 4 revascularization due to peripheral artery disease and 13 diagnoses of intermittent claudication.

Table 1 shows the combined, cardio- and cerebro-vascular events stratified by MD score classes. The number of combined events was the highest in subjects with score 0–1 (9.2%), lower in those with score 2–3 (5.0%) and the lowest in those with score 4–7 (2.7%). Similar rates were obtained considering cardio- and cerebro-VEs separately [1].

**Table 1:** Vascular events stratified according to the MD score.

MD Score	0-1 n (%)	2-3 n (%)	4-7 n (%)
Combined events (n = 215)	101 (9.2)	94 (5.0)	20 (2.7)
Cardiovascular events (n = 125)	58 (5.3)	56 (3.0)	11 (1.5)
Cerebrovascular events (n = 73)	32 (2.9)	33 (1.8)	8 (1.1)

**Fig. 1:**
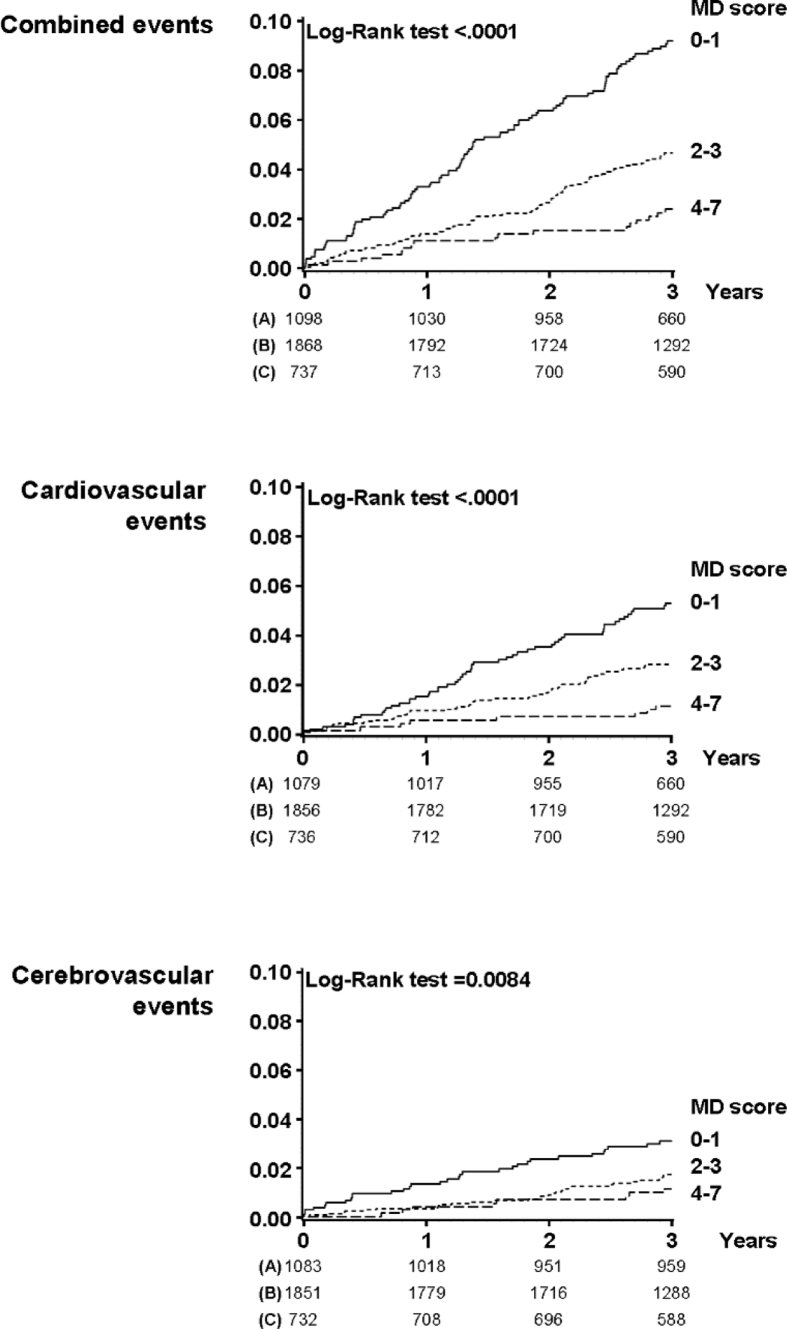
Kaplan-Meier incidence curves of combined, cardio and cerebro vascular events stratified by MD adherence score classes.

Fig. 1 specifies these results in detail, showing the Kaplan-Meier incidence curves of the combined endpoint, and of cardio- and cerebro-VEs, stratified by MD adherence score classes. Regardless of the endpoint considered, the rate of events was the highest in subjects with score 0–1, lower in those with score 2–3 and the lowest in those with score 4–7.

## 2 Experimental design, materials and methods

The IMPROVE was a multicenter, prospective cohort study including 3,703 patients (1,774 men, 1,929 women, aged 55–79 years) with ≥3 vascular risk factors, free from cardio- or cerebro-VEs [2], [3], [4]. Participants were recruited in 5 European countries and followed for 36 months. The combined endpoint is a composite of myocardial infarction, sudden cardiac death, angina pectoris, ischemic stroke, transient ischemic attack, new diagnosis of intermittent claudication or any surgical intervention or revascularization of coronary or peripheral arteries.

Cardiovascular events include acute myocardial infarction, angina pectoris, coronary angioplasty or bypass grafting and sudden cardiac death. Cerebrovascular events include ischemic stroke, transitory ischemic attack.

The MD adherence score was based on intake of 7 items: fruits, fish, wine, olive oil, meat, milk and eggs. For fruit or fish, high consumption (top tertile of their distributions*,* i.e. fruit ≥3 servings/day and fish >2 times/week) received one point, other intakes received 0 points; for meat, eggs or milk a low intake (bottom tertile of their respective distributions, i.e. meat <2 times/week, eggs ≤1 times/week, milk ≤3 dL/day) received one point. A predominant consumption of olive oil, rather than of other types of fat, and a moderate consumption of wine (1–2 glasses/day) also received one point each. Based on the scale obtained, score 0 indicates minimal adherence and score 7 maximal adherence to MD.
