# Supplementary material for: Data on the association between a simplified Mediterranean diet score and the incidence of combined, cardio and cerebro vascular events
Source: Data Brief. 2019 Feb 28;23:103789. doi: 10.1016/j.dib.2019.103789 (PMC6660565; doi:10.1016/j.dib.2019.103789)
Supplement: Multimedia component 1 [file mmc1.docx]

**AUTHOR DECLARATION TEMPLATE**

We wish to confirm that there are no known conflicts of interest associated with this publication.

On behalf of all co-authors:

Damiano Baldassarre, PhD.

Department of Medical Biotechnology and Translational Medicine, Università di Milano, Via Vanvitelli 32, 20129 - Milan, Italy and Centro Cardiologico Monzino, IRCCS, Via Parea 4, 20138 - Milan, Italy.

**Contact email:** [damiano.baldassarre@unimi.it](mailto:damiano.baldassarre@unimi.it); [damiano.baldassarre@ccfm.it](mailto:damiano.baldassarre@ccfm.it)
